# Magnetic-lens-generated polarized neutron beam with enhanced intensity and *Q* resolution for small-angle neutron scattering

**DOI:** 10.1107/S1600576725010246

**Published:** 2026-02-01

**Authors:** Kosuke Hiroi, Rintaro Nakabe, Takayuki Oku, Takayuki Kumada, Ryuhei Motokawa

**Affiliations:** a Materials Sciences Research Center Japan Atomic Energy Agency 2-4 Shirakata Tokai Ibaraki319-1195 Japan; b Japan Proton Accelerator Research Complex (J-PARC) Center Japan Atomic Energy Agency 2-4 Shirakata Tokai Ibaraki319-1195 Japan; Australian Centre for Neutron Scattering, ANSTO, Australia

**Keywords:** neutron magnetic lenses, polarization analysis, polarized small-angle neutron scattering

## Abstract

We installed a 30 mm-bore 3.2 m-long magnetic lens in the small-angle neutron scattering diffractometer (SANS-J) at the Japan Research Reactor 3 to focus the neutron beam near the sample position for measurements at *Q* > 0.1 nm^−1^. The focused beam at the sample position was over three times more intense than the standard non-focused beam, while the *Q* resolution was improved. The positive spin component of the neutron beam was focused near the sample, whereas the negative spin component was defocused and blocked by the slit just in front of the sample, producing a polarized beam. The 8 mm-diameter focusing polarized beam achieved a polarization of 0.95–0.96 and an intensity up to 22 times greater than that of a non-focusing polarized beam with the same collimation length. Using this beam with a remanent supermirror-coated spin analyser, we demonstrate that polarization analysis measurements of hydrogen-containing samples can be completed within tens of minutes.

## Abstract 2

While maintaining or even improving *Q* resolution, a long array of short-focal-length magnetic lenses, with zero transmission loss and no diffuse scattering, successfully polarized and enhanced the intensity of neutron beams for small-angle neutron scattering at momentum transfer *Q* > 0.1 nm^−1^.

## 1. Introduction

A lens is a common optical component, but it is used much less frequently in neutron diffractometers, except for small-angle neutron scattering (SANS) with monochromatic cold neutron sources (Radulescu, 2025 ▸; Kumada *et al.*, 2023*a* ▸; Barker *et al.*, 2022 ▸; Cremer *et al.*, 2020 ▸; Heller *et al.*, 2018 ▸; Wood *et al.*, 2018 ▸; Radulescu *et al.*, 2016 ▸; Heller *et al.*, 2014 ▸; Zhang *et al.*, 2014 ▸; Han *et al.*, 2013 ▸; Wignall *et al.*, 2012 ▸; Iwase *et al.*, 2011 ▸; Koizumi *et al.*, 2007 ▸; Oku *et al.*, 2007 ▸). Because the refractive indices of materials for thermal and cold neutron beams are close to 1, many biconcave lenses with small radii of curvature are required to focus the beams to long focal lengths, *f* (Choi *et al.*, 2000 ▸). In addition, because *f* is inversely proportional to the square of the neutron wavelength, λ, these lenses are only effective for monochromatic cold neutrons.

**Figure 1:**
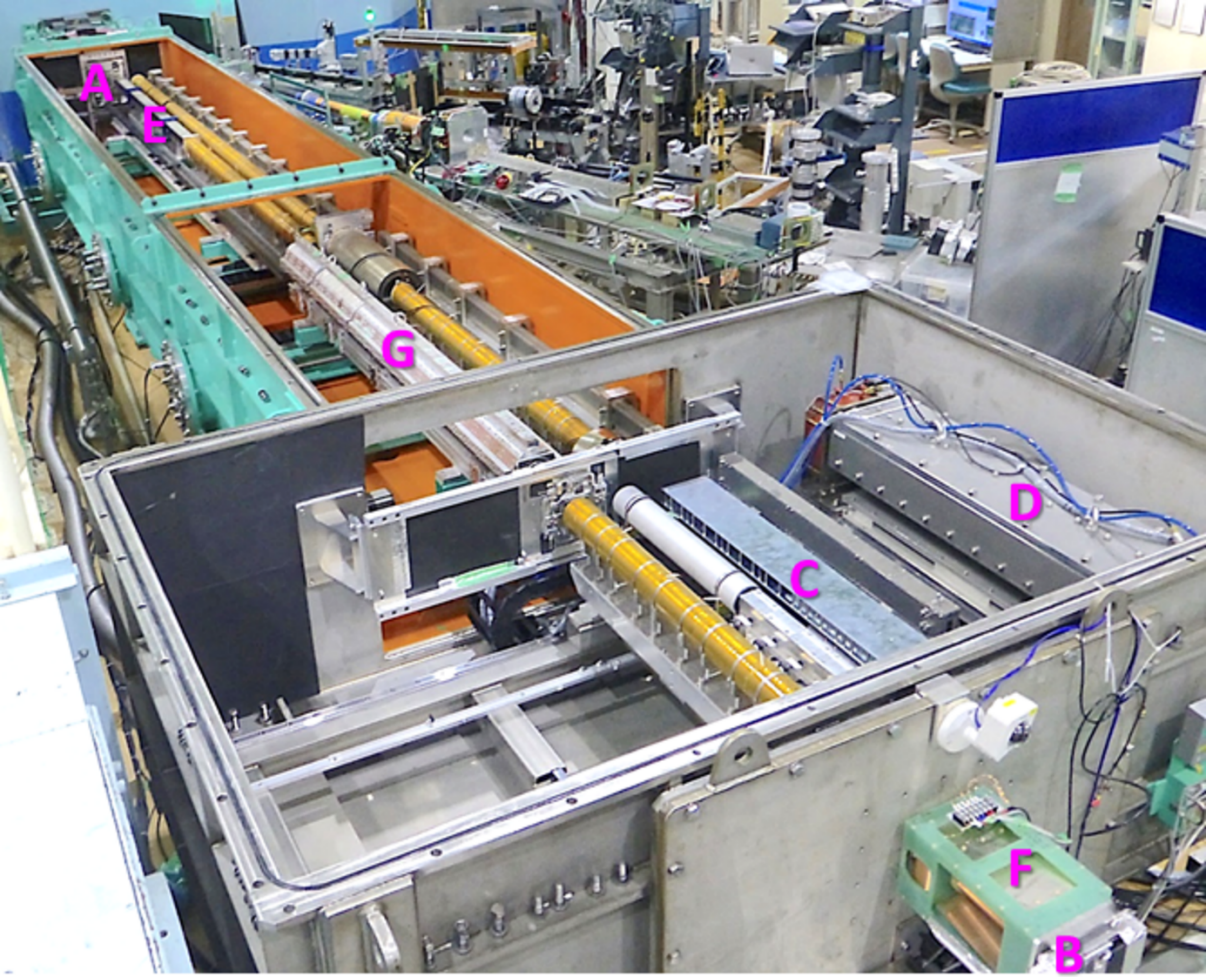
Photograph of the neutron optics in the SANS-J collimator. The components are (A) upstream four-jaw slit, (B) downstream slit, (C) MgF_2_ lenses, (D) preexisting magnetic lens, (E) supermirror polarizer, (F) gradient-RF neutron spin flipper and (G) extended magnetic lens.

Fig. 1 ▸ shows the neutron optics in the collimator of the 20 m-long small-angle neutron scattering diffractometer (SANS-J) at the Japan Research Reactor 3 (JRR-3) of the Japan Atomic Energy Agency (JAEA). Seventy biconcave MgF_2_ lenses, each with a 25 mm radius of curvature, are installed just in front of the sample. The lenses have a combined focal length of *f* = 4820 mm at λ = 0.65 nm. The lenses transfer the image of the neutron beam from the narrow upstream slit to the detectors at the downstream end of the diffractometer, enabling measurements of scattering down to a momentum transfer, *Q*, of 0.002 nm^−1^ (Kumada *et al.*, 2023*a* ▸). The neutron transmittance of the MgF_2_ lenses is 0.61 for a 15 mm-diameter neutron beam and decreases significantly for larger-diameter beams because the thickness of the lens increases as it moves away from the central axis. In addition, the MgF_2_ lenses produce diffuse scattering, which overlaps with the scattering signal from the sample (Kumada *et al.*, 2024 ▸). Therefore, tighter focusing cannot be achieved by installing more MgF_2_ lenses.

**Figure 2:**
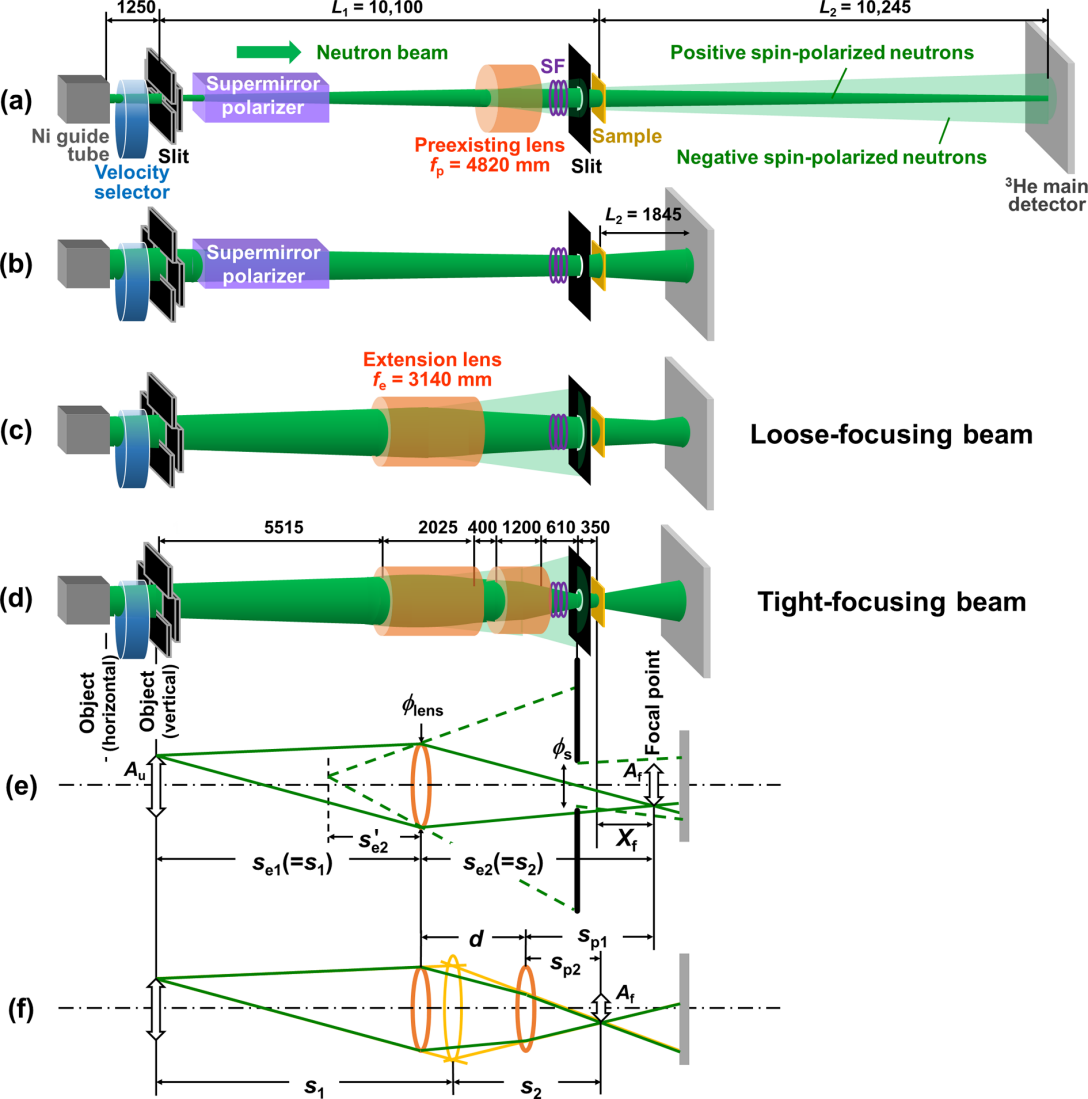
Conventional layouts for (*a*) the polarized focusing beam projected onto the detector at the downstream end (Oku *et al.*, 2007 ▸) and (*b*) the polarized non-focusing beam projected onto the detector at *L*_2_ = 1835 mm; new layouts for (*c*) the loose-focusing beam and (*d*) the tight-focusing beam; and conceptual drawings of the focusing and polarization of (*e*) the loose-focusing beam and (*f*) the tight-focusing beam. In the conventional layout for the unpolarized non-focusing beam, the supermirror polarizer in (*b*) is removed. The dashed lines in (*e*) indicate the trajectory of negative spin-polarized neutrons. The yellow lines and ellipse in (*f*) show the virtual lens, illustrating the combined focusing action of the preexisting and extended lenses.

A 1.2 m-long-sextupole magnetic neutron lens is also installed in SANS-J to focus polarized neutron beams onto the detector at the downstream end [Fig. 2 ▸ (*a*)]. This lens focuses and defocuses positive and negative spin-polarized neutron beams relative to the magnetic field direction, with *f* = +4820 and −4820 mm, respectively, at λ = 0.65 nm (Oku *et al.*, 2007 ▸). The magnetic lens can focus the positive spin-polarized neutrons entering the lens bore with a diameter, ϕ_lens_, of 30 mm, without transmission loss or diffuse scattering. However, the negative spin-polarized neutrons, some of which pass through the supermirror polarizer, are defocused by the lens, pass through the downstream slit just behind the lens and overlap with the scattering positive spin-polarized neutrons from the sample on the detector, producing background for scattering measurements. For this reason, we have recently used MgF_2_ lenses instead of the magnetic lens even for polarized neutron scattering measurements.

However, here we re-evaluate the advantages of the magnetic lens—namely, its lack of transmission loss and its relatively large bore diameter compared with the effective focusable diameter of the MgF_2_ lens—and propose a new layout employing an extended 3.2 m-long array of magnetic lenses with a focal length of approximately 2400 mm for scattering measurements at *Q* > 0.1 nm^−1^. The lenses focus positive spin-polarized neutrons from the upstream slit within the lens bore’s solid angle onto or near the sample position, rather than the detector [Figs. 2 ▸(*c*) and 2 ▸(*d*)]. Meanwhile, most of the defocusing negative spin-polarized neutrons are blocked by the downstream slit located just in front of the sample. As a result, the extended magnetic lens increases the neutron beam intensity at the sample position while simultaneously polarizing the beam.

The focusing beam provided by the extended magnetic lens is also useful for unpolarized neutron scattering measurements. Typically, the intensity of an unpolarized neutron beam is increased by installing Ni guide tubes in the collimator to shorten the collimation length for high-*Q* SANS measurements, but at the cost of an enlarged beam size at the detector. Since the detection area of the ^3^He main detector of SANS-J is only 0.4 m^2^, which is much smaller than that of other SANS diffractometers (Radulescu, 2025 ▸; Barker *et al.*, 2022 ▸; Cremer *et al.*, 2020 ▸; Heller *et al.*, 2018 ▸; Wood *et al.*, 2018 ▸; Radulescu *et al.*, 2016 ▸; Heller *et al.*, 2014 ▸; Zhang *et al.*, 2014 ▸; Han *et al.*, 2013 ▸; Wignall *et al.*, 2012 ▸), the increased beam size confines *Q*-resolved SANS signals to a very narrow *Q* range. Thus, the collimation length of SANS-J is not shortened even for measurements at *Q* > 0.1 nm^−1^ (Kumada *et al.*, 2023*a* ▸).

In contrast, beam focusing using the extended magnetic lens enhances the beam intensity while maintaining—or even decreasing—the beam size at the detector, making it suitable for diffractometers with small detectors like SANS-J. For unpolarized SANS measurements, a similar increase in beam intensity and decrease in beam size can be achieved simultaneously by moderately shortening the collimation length and decreasing the slit aperture. However, for polarized SANS measurements, the advantage of the extended magnetic lens becomes significant, as it produces a polarized neutron beam without the transmission loss associated with a supermirror polarizer. On the basis of simple geometrical-optics simulations of intensity enhancement and polarization, we report basic scattering data and demonstrate that polarization analysis measurements can be performed using a remanent supermirror-coated spin analyser.

## 2. Experimental

### 2.1. Optical layout

Figs. 2 ▸(*a*)–2 ▸(*d*) compare the conventional and new layouts for polarized neutron scattering at SANS-J. The incident neutron beam from the end of the 20 mm-wide 50 mm-high Ni guide tube is monochromated with a velocity selector (Kuroda Precision Industries Ltd, Japan). The monochromated beam, with λ = 0.65 nm and wavelength resolution Δλ/λ = 0.15, is collimated using a four-jaw slit (*A*_u_ = 20 mm square aperture) at *L*_1_ = 10100 mm upstream of the sample, and a downstream slit (ϕ_s_ = 8 or 14 mm circular aperture) located 350 mm in front of the sample, to measure the scattering with the detector at *L*_2_ downstream of the sample. Neutron spins are flipped using a gradient radio frequency (RF) neutron spin flipper (SF) downstream of the magnetic lenses.

In the conventional layout, the incident neutron beam is polarized using a 2500 mm-long Z-shaped Fe/Si supermirror polarizer (PN04-38, Swiss Neutronics). The polarized neutron beam is focused onto the ^3^He main detector at *L*_2_ = 10245 mm with the 1200 mm-long preexisting lens [Fig. 2 ▸(*a*)], or collimated by the upstream and downstream slits without the lens at *L*_2_ ≤ 3845 mm [Fig. 2 ▸(*b*)]. In the new layout, an extended magnetic lens, consisting of 500 and 1500 mm-long segments with ϕ_lens_ = 30 mm (Oku *et al.*, 2010 ▸) and a focal length *f*_e_ = 3140 mm, is installed. The loose-focusing beam [Fig. 2 ▸(*c*)] is designed to be focused near the ^3^He main detector at *L*_2_ = 1845 mm using the extended lens alone for high-intensity high-*Q*-resolution scattering measurements. The tight-focusing beam [Fig. 2 ▸(*d*)] is focused onto the sample position using both the extended magnetic lens and the preexisting lens, enabling high-intensity high-neutron-polarization (*P*_n_) scattering measurements.

Figs. 2 ▸(*e*) and 2 ▸(*f*) are conceptual drawings showing how the positive and negative spin-polarized neutrons are focused and defocused by the magnetic lenses. In this model, the object position in the horizontal direction is taken at the end of the 20 mm-wide Ni guide tube, located 1200 mm upstream of the upstream slit. This choice reflects the fact that neutrons do not propagate inward horizontally from the outer edge at the *A*_u_ = 20 mm upstream slit. The extended lens and the preexisting lens are each approximated as a single 30 mm-diameter thin lens, located at object distances *s*_e1_ and *s*_e1_ + *d* downstream from the object position, respectively. In the loose-focusing beam configuration, the positive spin-polarized neutrons form a square image with side length *A*_f_ = (*s*_e2_/*s*_e1_)*A*_u_ at the image distance *s*_e2_ = *s*_e1_*f*_e_/(*s*_e1_ − *f*_e_) downstream of the extended lens. The extended lens is positioned closer to the downstream slit than to the upstream slit to narrow the beam both at the focal point and at the downstream slit. Here, the slit width ϕ_s_ is 14 mm for general SANS measurements and 8 mm for measurements of small samples and for polarization analysis. In contrast, the negative spin-polarized neutrons are defocused downstream of the lens with a divergence of ϕ_lens_/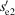, where 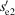 = *s*_e1_*f*_e_/(*s*_e1_ + *f*_e_) is the absolute virtual image distance upstream of the lens. While the intensity of the non-focusing beam is proportional to the solid angle subtended by the aperture of the downstream slit as seen from the object, the intensity of the focusing positive spin-polarized beam is proportional to the solid angle subtended by the lens bore with ϕ_lens_ from the object, multiplied by the transmission ratio of the downstream slit.

In the tight-focusing beam configuration, the preexisting lens transfers the image at the focal point of the extended lens to produce a square image with a side length *A*_f_ = (*s*_p2_/*s*_e1_)*A*_u_ at the image distance *s*_p2_ = *s*_p1_*f*_p_/(*s*_p1_ + *f*_p_) downstream of the preexisting lens, where *s*_p1_ = *s*_e2_ − *d* = *s*_e1_*f*_e_/(*s*_e1_ − *f*_e_) − *d* is the absolute virtual object distance downstream of the preexisting lens with the focal length *f*_p_ = 4820 mm. The combination of the extended lens and the preexisting lens can be represented as a single virtual lens that focuses the positive spin-polarized neutrons with the object distance *s*_1_ = [*s*_e1_/(*s*_e1_ + *s*_p2_)](*s*_e1_ + *d* + *s*_p2_), the image distance *s*_2_ = [*s*_p2_/(*s*_e1_ + *s*_p2_)](*s*_e1_ + *d* + *s*_p2_) and the focal length *f* = *s*_1_*s*_2_/(*s*_1_ + *s*_2_) and defocuses the negative spin-polarized neutrons with the virtual focal length of −*s*_1_*s*_2_/(*s*_1_ + *s*_2_).

Table 1 ▸ compares the calculated object length *s*_1_, image lengths *s*_2_ and 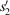 for the positive and negative spin-polarized neutrons, respectively, and focal point distance *X*_f_ from the sample. This table also compares the intensity enhancement of the loose- and tight-focusing beams relative to the unpolarized non-focusing beam, ɛ, which is calculated by using the geometric model in Figs. 2 ▸(*e*) and 2 ▸(*f*). *P*_n_ was obtained from the transmission ratios of the downstream slit for the positive and negative spin-polarized neutrons. Both ɛ and *P*_n_ of the tight-focusing beam are higher than those of the loose-focusing beam because of the tighter focusing of the positive spin-polarized neutrons and wider defocusing of the negative spin-polarized neutrons at the downstream slit. ɛ of the loose-focusing beam with ϕ_s_ = 14 mm is equal to that with ϕ_s_ = 8 mm because the beam size at the downstream slit exceeds ϕ_s_; thus, the intensity of both the non-focusing and loose-focusing beams is proportional to 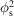. In contrast, ɛ of the tight-focusing beam with ϕ_s_ = 14 mm is smaller than that with ϕ_s_ = 8 mm because the beam size becomes smaller than ϕ_s_ = 14 mm; thus, the intensity of the tight-focusing beam is no longer proportional to 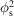.

We measured the beam images at the sample position using the neutron CCD camera (100 mm × 100 mm, Neutron Optics Grenoble, France) and at the detector position using the highly position-sensitive photomultiplier tube (PS-PMT; R3239, Hamamatsu, Japan) that is generally used for ultra-small-angle neutron scattering measurements (Hirota *et al.*, 2005 ▸; Koizumi *et al.*, 2007 ▸).

### 2.2. Polarization analysis

**Figure 3:**
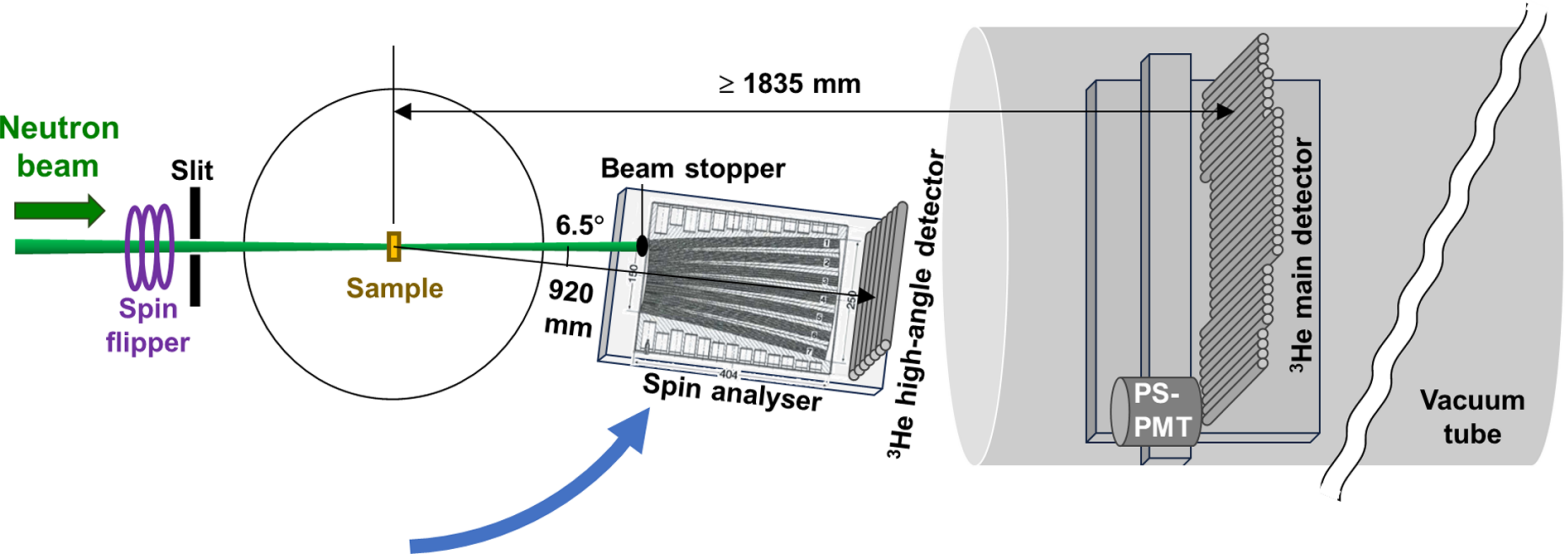
Layout for the polarization analysis measurement. The ^3^He high-angle detector with the remanent supermirror-coated spin analyser was moved in front of the vacuum tube containing the ^3^He main detector and the PS-PMT (Iwase *et al.*, 2007 ▸).

The neutron polarization analysis measurements were performed using the tight-focusing beam with *A*_u_ = 20 mm and ϕ_s_ = 8 mm, and a spin analyser consisting of remanent FeCoV/TiN_*x*_ supermirrors: 2.5*Q*_c_ concave and 1.5*Q*_c_ convex, where *Q*_c_ is the critical *Q* for total reflection of the Ni mirror, with detection solid angle of 0.07 sr (PN03-25, SwissNeutronics) (Böni *et al.*, 1999 ▸; Iwase *et al.*, 2007 ▸; Noda *et al.*, 2013 ▸). The scattering neutrons transmitted through the spin analyser were measured by a high-angle detector consisting of a horizontal array of 31 position-sensitive detectors, each 8 mm in diameter and filled with 0.5 MPa ^3^He gas (E6882-300, J5, Canon, Japan), at *L*_2_ = 920 mm (Fig. 3 ▸). For the polarization measurement of the non-scattered direct beam, the 20 mm-diameter beam stopper in front of the spin analyser was removed. For the polarization analysis measurement, the beam stopper was placed at the left end of the spin analyser. The samples measured were 1 mm-thick H_2_O, 1 mm-thick silver behenate powder (>95.0%, Tokyo Chemical Industry Co. Ltd, Japan) and 3 mm-thick V_0.96_Ni_0.04_ alloy (Taiyo Koko Co. Ltd, Japan), which has a coherent scattering cross section of 0.

Because the positions at which the scattered neutrons were detected were shifted horizontally according to the reflection at the curved supermirrors in the spin analyser, the ring-shaped Bragg peaks of silver behenate (see below) were used to calibrate *Q* of the scattered neutrons at each detector pixel. The sensitivity distribution of the detector was calibrated by the scattering intensity of a 1.2 mm-thick polyethyl­ene sample. The scattering intensity of each sample was converted to absolute units based on the scattering intensity of the glassy carbon standard sample (Thermo Fisher Scientific Inc., USA).

## 3. Results and discussion

### 3.1. Comparison with unpolarized non-focusing beam

**Figure 4:**
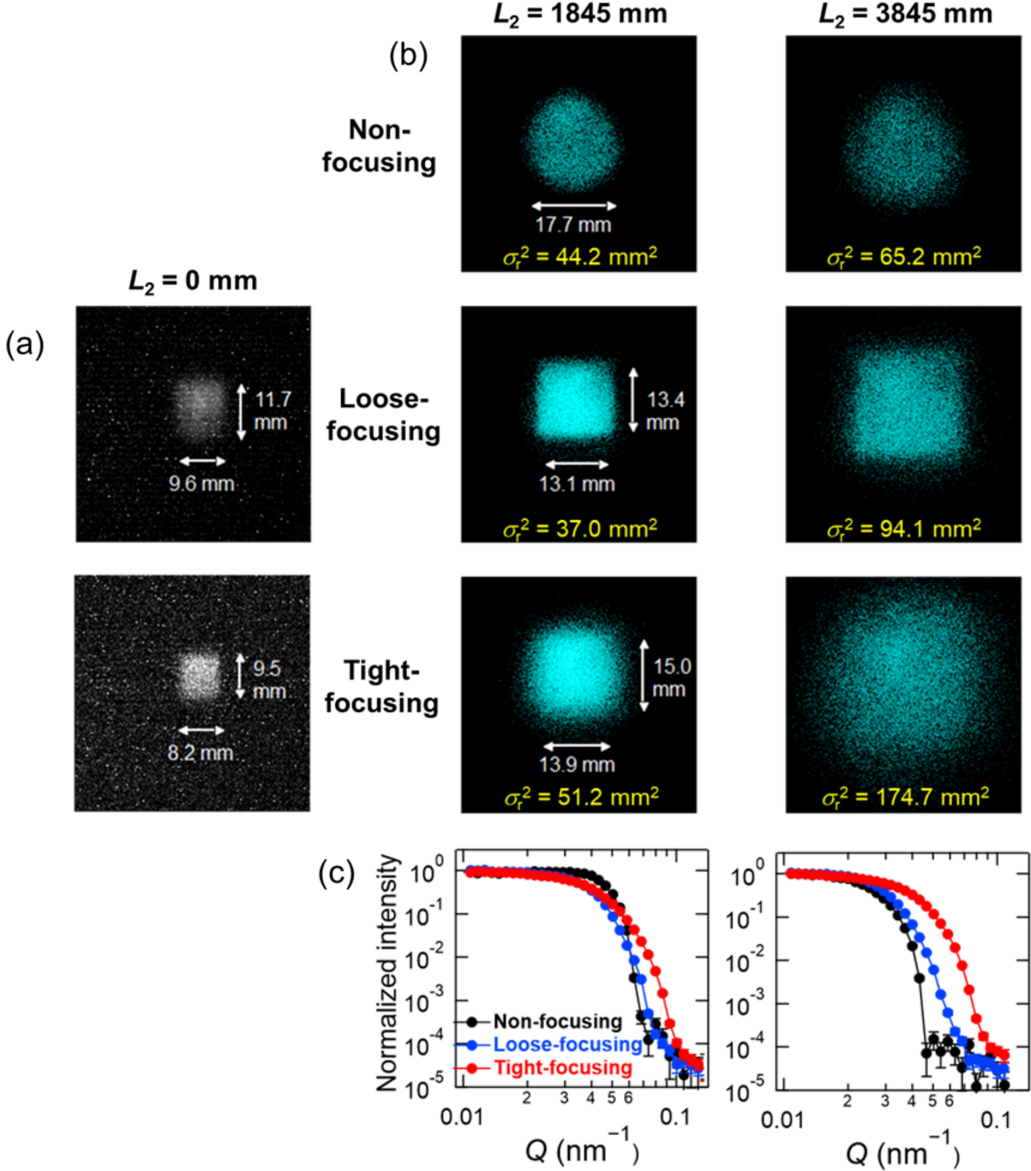
(*a*) CCD images of the focusing beams at the sample position without the downstream slit, (*b*) PS-PMT images of the unpolarized non-focusing beam and the focusing beams with ϕ_s_ = 14 mm at *L*_2_ = 1845 and 3845 mm, and (*c*) radially averaged intensity distribution obtained from (*b*). *A*_u_ = 20 mm was used for all the measurements.

Fig. 4 ▸(*a*) shows CCD images of the loose- and tight-focusing beams at the sample position (*L*_2_ = 0 mm) without a downstream slit. Fig. 4 ▸(*b*) compares the PS-PMT images of the non-focusing and focusing beams with ϕ_s_ = 14 mm at *L*_2_ = 1845 and 3845 mm. At *L*_2_ = 1845 mm, the loose-focusing beam had a clearer square image than the tight-focusing beam, indicating that the focal point of the loose-focusing beam was closer to the detector than that of the tight-focusing beam. Compared with the non-focusing beam, the statistical variance σ_r_^2^ was smaller for the loose-focusing beam and larger for the tight-focusing beam. At *L*_2_ = 3845 mm, the square image in both the loose- and tight-focusing beams became less distinct due to the increased distance from the focal point to the detector. The σ_r_^2^ value of the loose-focusing beam was somewhat larger than that of the non-focusing beam, while that of the tight-focusing beam was considerably larger due to the larger beam divergence. Fig. 4 ▸(*c*) shows the radially averaged normalized intensity distribution obtained from the images in Fig. 4 ▸(*b*). Whereas the non-focusing, loose-focusing and tight-focusing beams are all similar at *L*_2_ = 1845 mm, the loose- and tight-focusing beams became broader than the non-focusing beam at *L*_2_ = 3845 mm. These results indicate that the technique of beam focusing close to the sample position can be used for high-*Q* SANS measurements only, where *L*_2_ < *s*_2_.

The 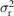 values of the non-focusing beam at *L*_2_ = 1845 and 3845 mm were very close to those calculated using the analytical model presented later in this paper (44.66 and 64.5 mm^2^, respectively). The tight-focusing beam produced a clear rectangular image whose widths in the horizontal and vertical directions were close to *A*_f_ in Table 1 ▸, indicating that the beam focused very close to the sample position as expected. However, the loose-focusing beam produced a blurred rectangular image whose widths were notably smaller than *A*_f_ in Table 1 ▸, and the image at *L*_2_ = 0 mm is smaller than that at *L*_2_ = 1845 mm. These results indicate that the extended magnetic lens produces a tighter focus than calculated, with the focal point located closer to the sample than to the detector. The single thin-lens model may have underestimated the focusing power of the extended lens.

**Figure 5:**
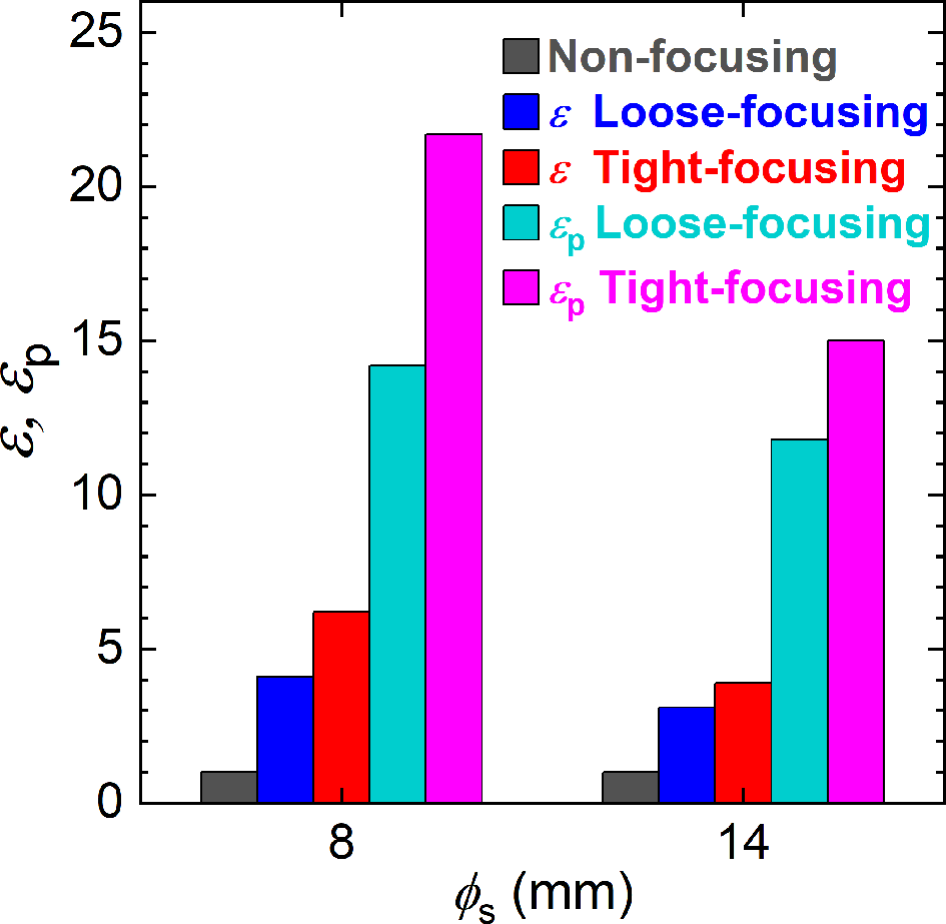
Intensity enhancements ɛ of the focusing beams relative to the unpolarized non-focusing beam. The enhancement ɛ_p_ relative to the polarized focusing beam, as shown in Table 2 ▸, is also plotted.

The intensity enhancement ɛ of the focusing beams relative to the unpolarized non-focusing beam, obtained from the PS-PMT image measurements, is plotted in Fig. 5 ▸. The trend of ɛ values agrees with the calculated results in Table 1 ▸ except for the loose-focusing beam with ϕ_s_ = 8 mm, whose value was larger than the calculated value. This discrepancy probably arises because, as discussed above, the beam was more tightly focused than calculated.

**Figure 6:**
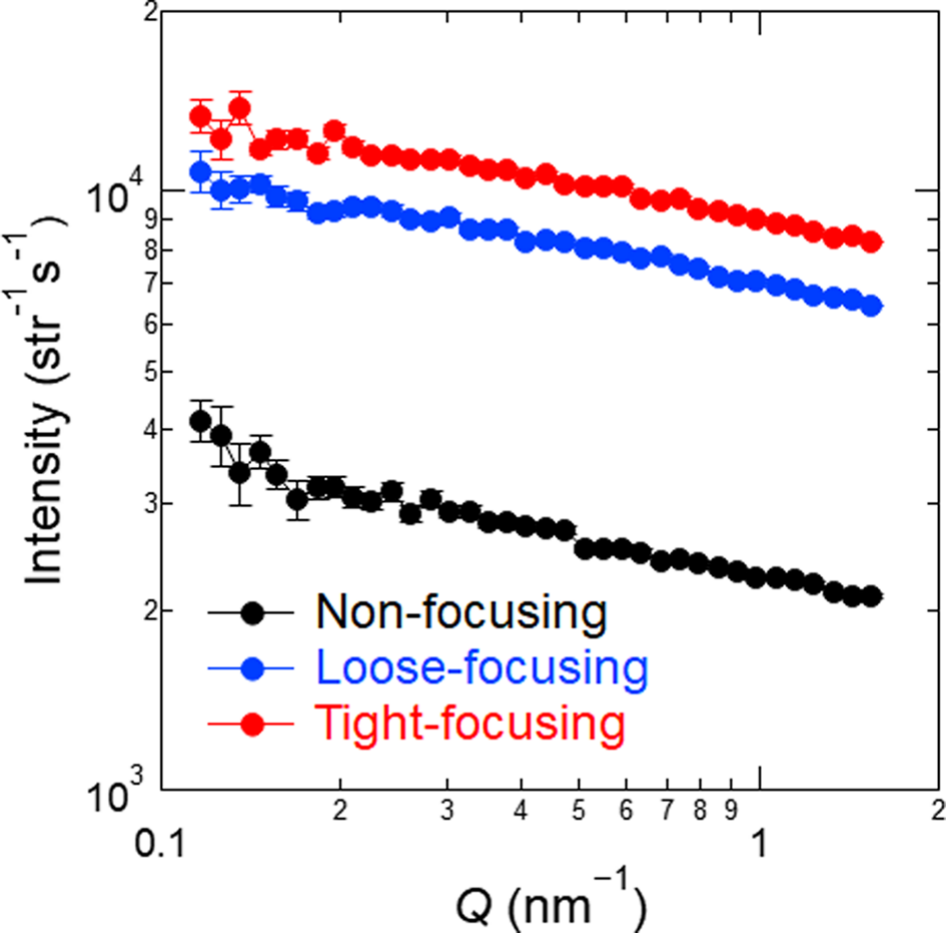
Intensity distribution of the non-focusing and focusing beams with *A*_u_ = 20 mm and ϕ_s_ = 14 mm at *L*_2_ = 1845 mm, as measured by the ^3^He main detector.

Fig. 6 ▸ compares the intensity distributions of the non-focusing and focusing beams at *Q* > 0.1 nm^−1^ measured with the ^3^He main detector at *L*_2_ = 1845 mm. The intensity distribution of the non-focusing beam in this *Q* range was attributed to the air scattering of the beam around the sample position (Kumada *et al.*, 2024 ▸). The intensity of the focusing beams increased proportionally to that of the non-focusing beam by ɛ, indicating that parasitic scattering from the magnetic lenses was not generated or at least its intensity was much smaller than the intensity of the air scattering.

**Figure 7:**
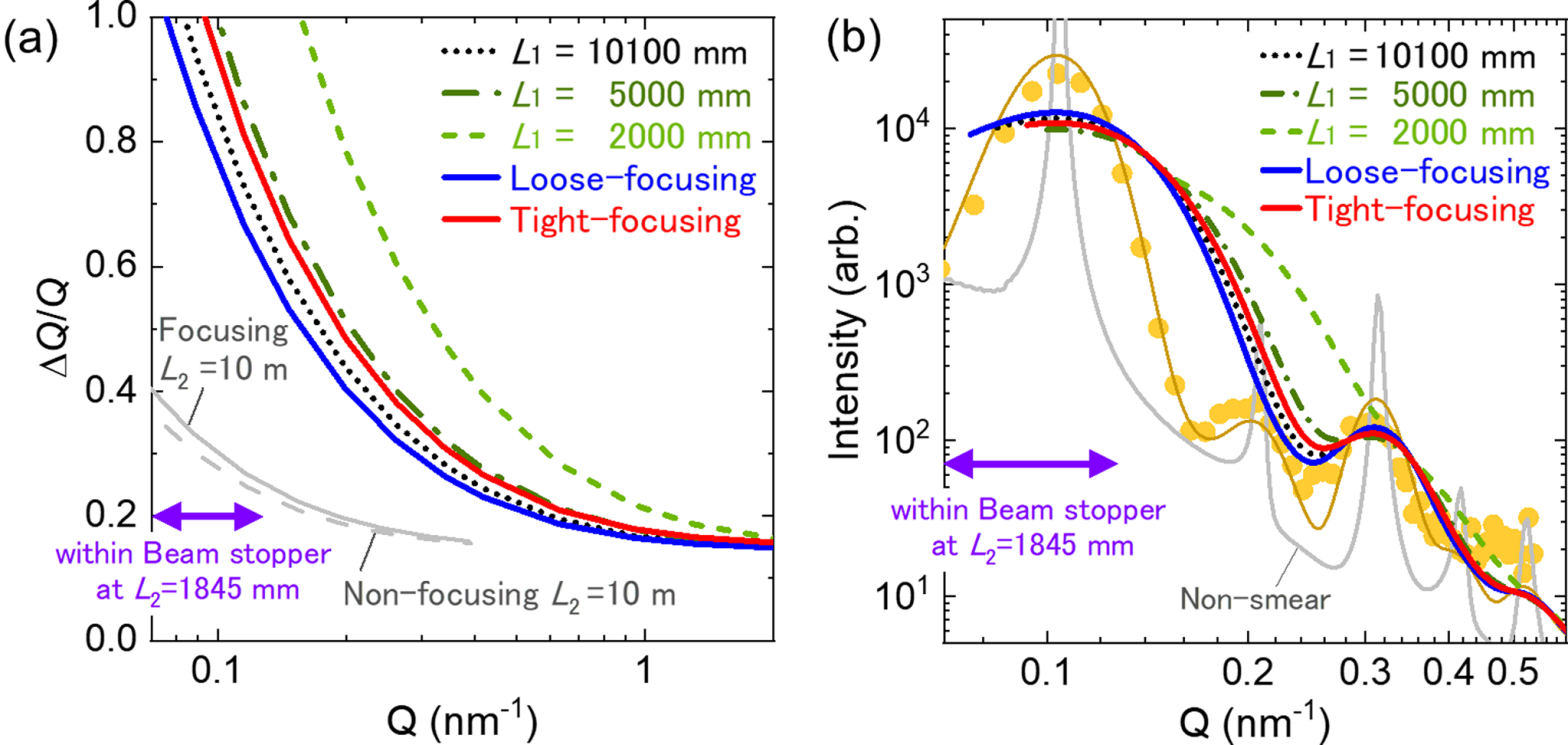
(*a*) Δ*Q*/*Q* as a function of *Q* for the loose- and tight-focusing beams and the collimator-shortened non-focusing beam with *A*_u_ = 20 mm, ϕ_s_ = 14 mm and *L*_2_ = 1845 mm. The grey dashed and solid lines correspond to the non-focusing beam with ϕ_s_ = 8 mm and the focusing beam with ϕ_s_ = 14 mm, respectively, at *L*_2_ = 10245 mm. The curves were simulated using the experimentally obtained 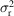 values in Fig. 4 ▸(*b*) for the non-focusing beam with *L*_1_ = 10100 mm, and for the loose- and tight-focusing beams. For the other beam configurations, the 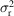 values calculated with equations (2) and (3) were used. (*b*) SANS profile of the microphase-separated lamellar poly(styrene-*b*-isoprene) cast film (yellow symbols) obtained using the non-focusing beam with *A*_u_ = 20 mm, ϕ_s_ = 8 mm, *L*_1_ = 10100 mm and *L*_2_ = 5000 mm (Noda *et al.*, 2011 ▸). The plot also shows simulated SANS curves using Δ*Q*/*Q* in (*a*) for the experimental beam (yellow narrow line); the non-focusing beam with *A*_u_ = 20 mm, ϕ_s_ = 14 mm, *L*_1_ = 10100, 5000 and 2000 mm, and *L*_2_ = 1845 mm (dotted, dash–dotted and dashed lines); and the loose- and tight-focusing beams with *L*_2_ = 1845 mm (solid lines), down to where Δ*Q*/*Q* = 1. The double-ended purple arrow shows the *Q* range where signals are hidden by the beam stopper of SANS-J at *L*_2_ = 1845 mm.

Generally, the collimation length *L*_1_ is shortened by installing Ni guide tubes in the upstream section of the collimator, so that it matches the sample-to-detector distance *L*_2_. The neutron beam is transported through the Ni guide tubes without significant intensity loss. The beam intensity increases because the solid angle subtended by the downstream slit, as viewed from the slit at the end of the Ni guide tube, is larger than that seen from the slit at the upstream end of the collimator. However, the Δ*Q*/*Q* obtained with the *L*_1_ = *L*_2_ = 2 m layout [Fig. 7(*a*)] is so poor that the SANS curves obtained with the layout do not coincide with the curve with the *L*_1_ = *L*_2_ = 10 m layout in the overlapped *Q* range between 0.1 and 0.4 nm^−1^, resulting in a decrease in structural analysis accuracy. This issue is particularly problematic for SANS diffractometers with small detectors, such as SANS-J, because the *Q* range covered in a single measurement at each *L*_2_ is severely limited by the small detection area of the detector. Δ*Q*/*Q* must be sufficiently small to ensure overlap of SANS curves measured at different *L*_2_ settings within the overlapping *Q* range.

Here, we quantitively compare the intensity enhancements and the smearing of SANS curves between the focusing beams and the collimator-shortened non-focusing beam. The *Q* resolution was calculated using the standard *Q* variance formula (Hammouda & Mildner, 2007 ▸):
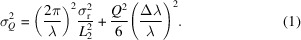
Here, for the non-focusing beam, 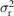 is given by
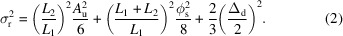
For the focusing beam, if ϕ_lens_ < ϕ_s_*s*_2_/*X*_f_,
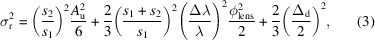
and, if ϕ_lens_ > ϕ_s_*s*_2_/*X*_f_,
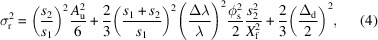
as long as the focal point is located at the detector. In these equations, Δ_d_ = 7 mm is the spatial resolution of the ^3^He main detector. Anisotropy of Δ_d_ and the gravity effect were neglected. The prefactor of the first term on the right side of equations (2), (3) and (4) differs from that given by Hammouda & Mildner (2007 ▸), as it has been modified for a rectangular first slit. Δ*Q*/*Q* is obtained from 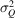:
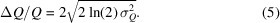


Since both the loose- and tight-focusing beams with ϕ_s_ = 14 mm, where ϕ_lens_ < ϕ_s_*s*_2_/*X*_f_, have the focal point in front of the detector, 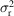 and Δ*Q*/*Q* of these beams at *L*_2_ = 1845 mm are larger than those calculated using equations (3) and (5).

In SANS-J, scattering curves are typically measured using the main detector positioned at *L*_2_ = 1845 and 10245 mm. At *L*_2_ = 1845 mm, while scattered neutrons with *Q* > 1.2 nm^−1^ can be detected without being blocked by a 35 mm-diameter beam stopper, the practically accessible minimum *Q* is 0.2 nm^−1^. At *L*_2_ = 10245 mm, the accessible maximum *Q* is about 0.3 nm^−1^, which is limited by the detector size. For accurate structure analysis, the *Q* resolution of the scattering curve measured at *L*_2_ = 1845 mm must be sufficiently high to ensure good overlap with the curve obtained at *L*_2_ = 10245 mm within the overlapping *Q* range of 0.2–0.3 nm^−1^.

Fig. 7 ▸(*a*) compares Δ*Q*/*Q* as a function of *Q* for the focusing beams with that for the collimator-shortened non-focusing beam (*A*_u_ = 20 mm, ϕ_s_ = 14 mm and *L*_2_ = 1845 mm). For the collimator-shortened non-focusing beam, decreasing *L*_1_ increases the beam intensity proportionally to 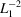, but at the cost of *Q* resolution. As shown in Fig. 7 ▸(*b*), the degradation of *Q* resolution leads to smearing of the SANS curve, which is particularly problematic for analyses of structured curves. With *L*_1_ = 2000 mm, the distinct modulation of the SANS curve of a microphase-separated lamellar cast film, obtained using the non-focusing beam with *L*_1_ = 10100 mm and *L*_2_ = 5000 mm (Noda *et al.*, 2011 ▸), is smeared out. We consider that the beam with *L*_1_ = 5000 mm provides a reasonable compromise between ɛ and Δ*Q*/*Q* within the adjustable layout of SANS-J (ɛ = 4 at a 17% increase in Δ*Q*/*Q* at *Q* = 0.2 nm^−1^ compared with *L*_1_ = 10100 mm). These values are close to those of the tight-focusing beam (ɛ = 3.9 with an 11% increase). The loose-focusing beam achieved ɛ = 3.1 with a 4% decrease in Δ*Q*/*Q*. Similar performance (ɛ = 2.8 with identical Δ*Q*/*Q*) can be obtained with the non-focusing beam with *L*_1_ = 4000 mm and ϕ_s_ = 9.2 mm (not shown). Thus, the focusing beams achieve an intensity enhancement while maintaining, or even slightly decreasing, Δ*Q*/*Q* in a manner similar to that of the non-focusing beam when *L*_1_ and ϕ_s_ are reduced.

### 3.2. Comparison with polarized non-focusing beam

The focusing beams offer a distinct advantage for polarized neutrons, because the beams are polarized without the supermirror polarizer whose transmission is 0.3. Table 2 ▸ shows the intensity and intensity enhancement against the polarized non-focusing beam, ɛ_p_, and *P*_n_*P*_ana_, as measured by the high-angle detector behind the spin analyser with polarization analysis efficiency *P*_ana_. The maximum values of ɛ_p_ were 21.7 and 15.0 for the tight-focusing beam with ϕ_s_ = 8 and 14 mm, respectively. *P*_n_*P*_ana_ was determined from the intensities of the direct beam when the SF was on, *I*_0,on_, and off, *I*_0,off_: *P*_n_*P*_ana_ = |*I*_0,off_ − *I*_0,on_|/(*I*_0,off_ + *I*_0,on_). The *P*_n_*P*_ana_ value of the polarized non-focusing beam was 0.86 at ϕ_s_ = 8 mm and 0.88 at ϕ_s_ = 14 mm, consistent with the previously reported value (Noda *et al.*, 2013 ▸). Substituting the reported value of *P*_n_ = 0.99 for the supermirror polarizer (Oku *et al.*, 2007 ▸; Noda *et al.*, 2013 ▸) into *P*_n_*P*_ana_ gave *P*_ana_ values of 0.87 and 0.89 at ϕ_s_ = 8 and 14 mm, respectively. The *P*_n_ values in parentheses were obtained by substituting the *P*_ana_ value into *P*_n_*P*_ana_. The *P*_n_ values for the tight-focusing beam were slightly smaller than *P*_n_ obtained by the supermirror polarizer, but high enough for polarized neutron scattering and polarization analysis measurements.

### 3.3. Polarization analysis measurements

**Figure 8:**
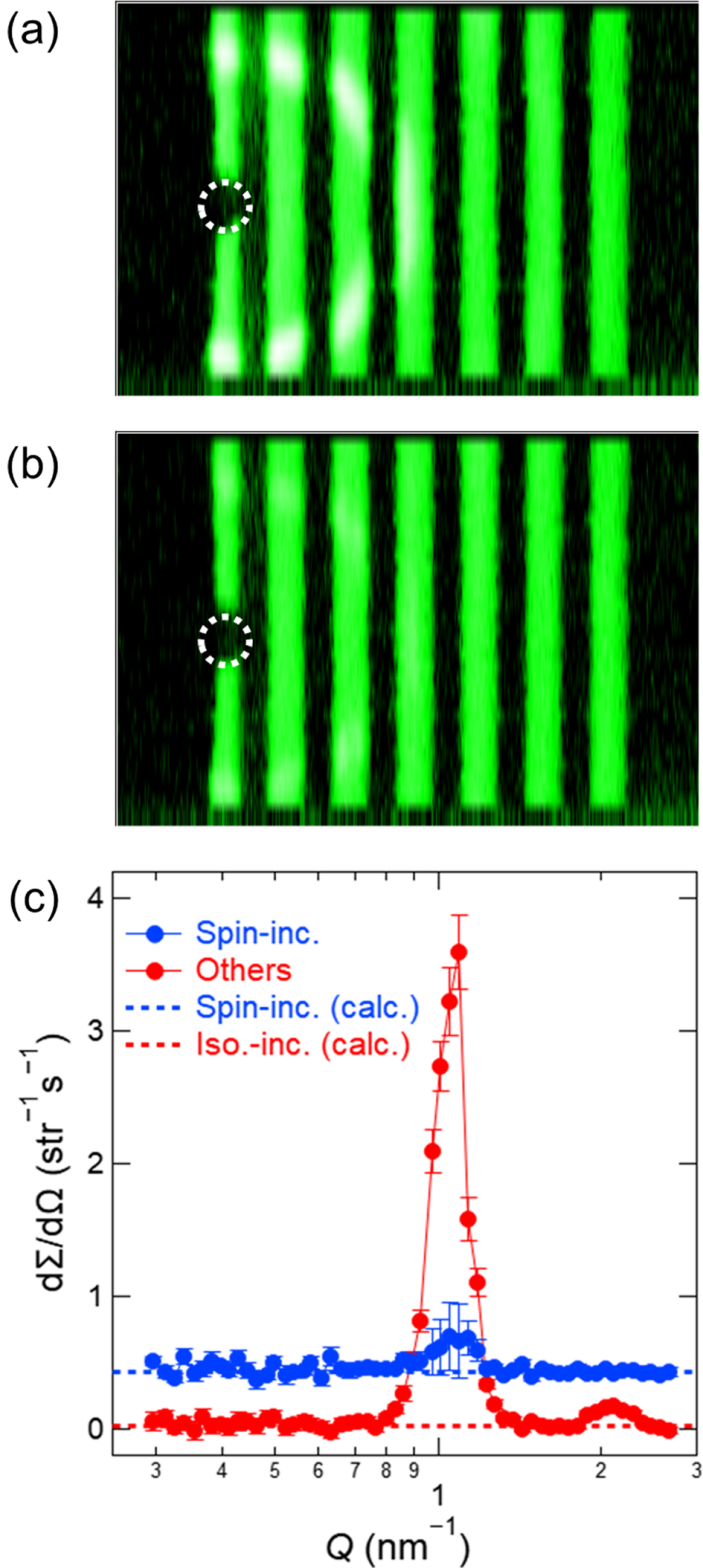
Two-dimensional polarized neutron scattering images of the silver behenate sample measured with the ^3^He high-angle detector behind the spin analyser for 1800 s each, with (*a*) SF off and (*b*) SF on. The dashed circle indicates the position of the beam stopper. (*c*) (dΣ/dΩ)_s-inc_(*Q*) and (dΣ/dΩ)_others_(*Q*) were obtained from (*a*) and (*b*) using *p* = 0.598. The dashed lines indicate the calculated spin- and isotope-incoherent differential scattering cross sections.

Figs. 8 ▸(*a*) and 8 ▸(*b*) show two-dimensional scattering images of the silver behenate sample measured by the ^3^He high-angle detector behind the spin analyser, with SF off and on. Coherent diffraction peaks from the silver behenate sample were clearly observed with SF off but significantly weaker with SF on. The differential scattering cross sections, dΣ/dΩ, were obtained by circularly averaging these images. Assuming a spin-flipper efficiency of 1, the spin-flip differential scattering cross section, (dΣ/dΩ)_SF_(*Q*), and non-spin-flip differential scattering cross section, (dΣ/dΩ)_NSF_(*Q*), were determined from (dΣ/dΩ)_on_(*Q*), (dΣ/dΩ)_off_(*Q*) and *P*_n_*P*_ana_ as
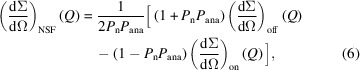

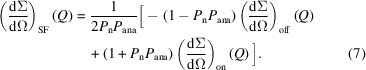
The spin-incoherent differential scattering cross section, (dΣ/dΩ)_s-inc_(*Q*), and the sum of the coherent and isotope-incoherent differential scattering cross sections, (dΣ/dΩ)_others_(*Q*), were given by
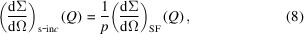

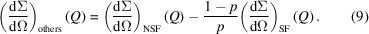
The spin-flipping probability for spin-incoherent scattering is *p* = 2/3 when a sample undergoes only a single scattering event, and it approaches 0.5 as the fraction of multiple-scattering events increases. We used *p* for each sample as obtained by substituting the spin-incoherent cross section of each element (Dianoux & Lander, 2003 ▸) into the general-purpose Monte Carlo simulation code *PHITS* (Sato *et al.*, 2024 ▸; Iwamoto *et al.*, 2022 ▸) to separate (dΣ/dΩ)_s-inc_(*Q*) and (dΣ/dΩ)_others_(*Q*). Fig. 8 ▸(*c*) shows (dΣ/dΩ)_s-inc_(*Q*) and (dΣ/dΩ)_others_(*Q*), which were obtained from Figs. 8 ▸(*a*) and 8 ▸(*b*) and equations (6)–(9). The diffraction peaks were clearly observed in (dΣ/dΩ)_others_(*Q*), with no plateau signal, as expected from the calculated isotope-incoherent differential scattering cross section. Conversely, a plateau consistent with the calculated spin-incoherent differential scattering cross section was observed in (dΣ/dΩ)_s-inc_(*Q*).

Contrary to our expectation, the diffraction peak was not eliminated from (dΣ/dΩ)_s-inc_(*Q*). We attribute this to the fact that the *P*_ana_ value for the scattered neutrons was slightly lower than that for the non-scattered direct beam used in equations (6) and (7). Because of the collimation geometry, the spin analyser exhibits the highest spin-analysing performance for neutrons scattered from the sample centre, but reduced performance for those scattered from off-centre regions (Böni *et al.*, 1999 ▸). In fact, (dΣ/dΩ)_s-inc_(*Q*) and (dΣ/dΩ)_others_(*Q*) determined using the beam with ϕ_s_ = 14 mm deviated more from the calculated values than those obtained with the beam with ϕ_s_ = 8 mm (data not shown). Thus, although the use of a narrower beam could in principle allow a more accurate determination of (dΣ/dΩ)_s-inc_(*Q*) and (dΣ/dΩ)_others_(*Q*), the significant loss of scattering intensity makes this approach impractical.

**Figure 9:**
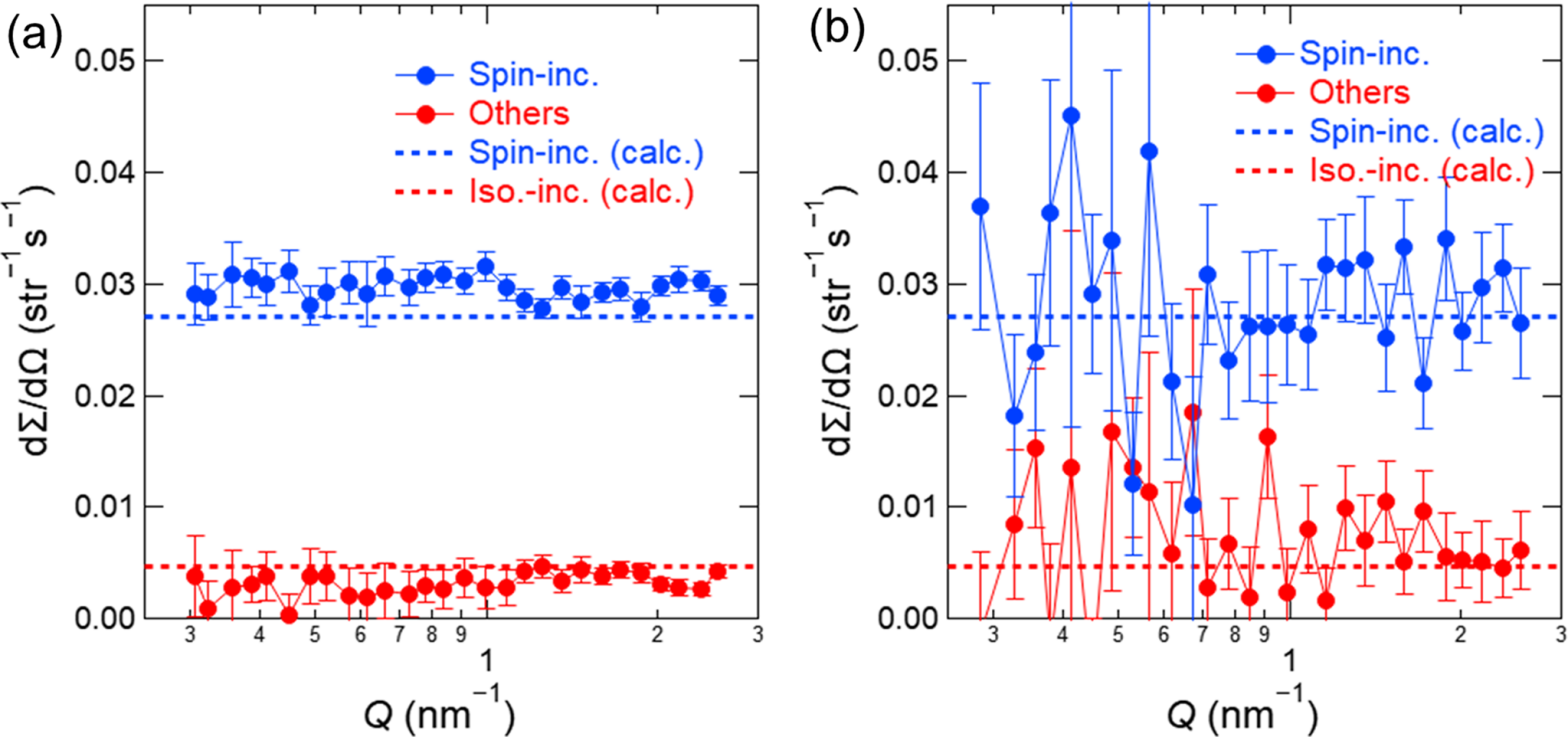
(dΣ/dΩ)_s-inc_(*Q*) and (dΣ/dΩ)_others_(*Q*) of V_0.96_Ni_0.04_ obtained from (dΣ/dΩ)_on_(*Q*) and (dΣ/dΩ)_off_(*Q*) measured with (*a*) the tight-focusing layout for 7200 s each and (*b*) the non-focusing layout for 3600 s each using *p* = 0.65. The dashed lines indicate the calculated spin- and isotope-incoherent differential scattering cross sections.

Fig. 9 ▸(*a*) shows (dΣ/dΩ)_s-inc_(*Q*) and (dΣ/dΩ)_others_(*Q*) of V_0.96_Ni_0.04_, which were obtained from (dΣ/dΩ)_SF_(*Q*) and (dΣ/dΩ)_NSF_(*Q*). Since this sample exhibited no coherent scattering, (dΣ/dΩ)_others_(*Q*) corresponds solely to isotope-incoherent scattering. The values of (dΣ/dΩ)_s-inc_(*Q*) and (dΣ/dΩ)_others_(*Q*) agree with the calculated spin- and isotope-incoherent differential scattering cross sections. These results demonstrate that SANS-J can determine spin- and isotope-incoherent differential scattering cross sections down to 0.005 cm^−1^ within its margin of error. Fig. 9 ▸(*b*) shows the data obtained with the non-focusing beam instead of the tight-focusing beam. The signal-to-noise ratio was too low to determine the differential cross sections. The remarkable improvement could not be expected even if the measurement time were set to be the same as that in Fig. 9 ▸(*a*). This result indicates that the detector behind the spin analyser can measure neutrons from only a limited region of the sample and that the acceptable solid angle of the spin analyser is very small (0.07 sr). The intensity enhancement using the tight-focusing beam enabled the polarization analysis of the sample.

**Figure 10:**
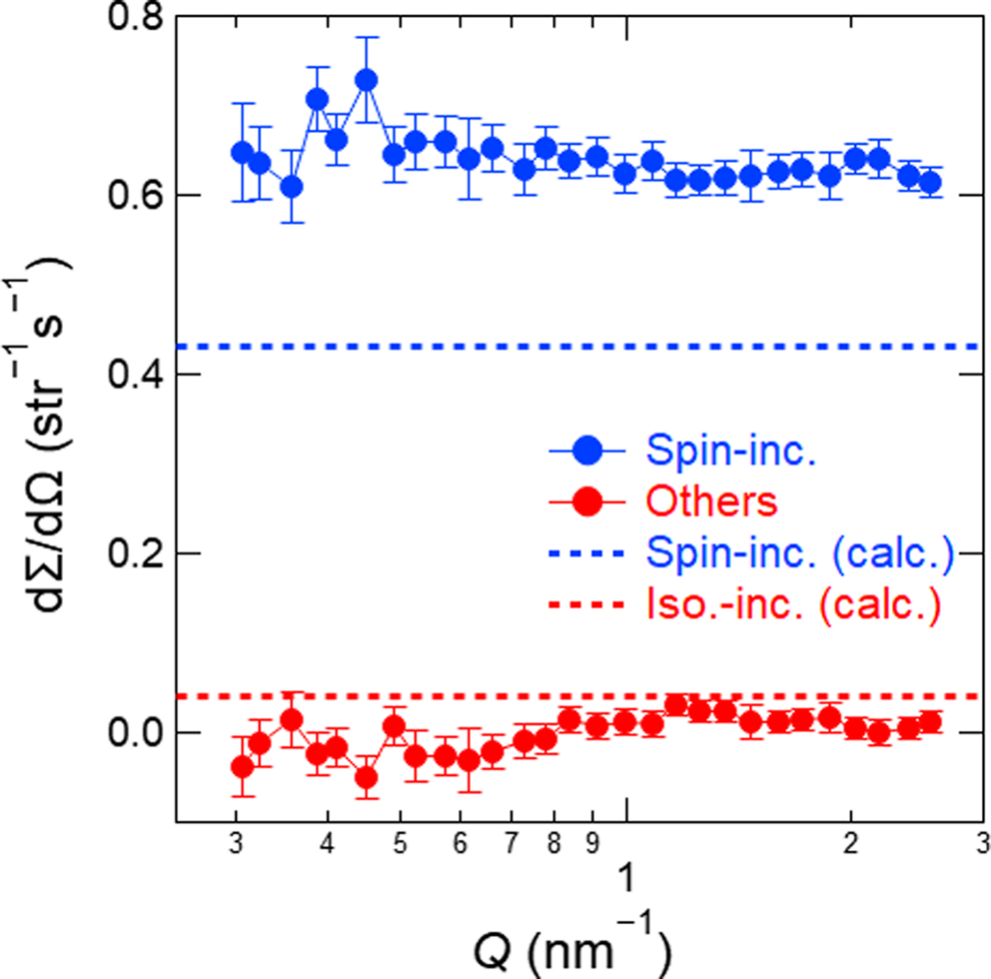
(dΣ/dΩ)_s-inc_(*Q*) and (dΣ/dΩ)_others_(*Q*) of the H_2_O sample obtained from (dΣ/dΩ)_on_(*Q*) and (dΣ/dΩ)_off_(*Q*) measured for 1800 s each, using *p* = 0.60. The dashed lines indicate the calculated spin- and isotope-incoherent differential scattering cross sections.

Fig. 10 ▸ shows (dΣ/dΩ)_s-inc_(*Q*) and (dΣ/dΩ)_others_(*Q*) of the 1 mm-thick H_2_O sample. Due to multiple scattering, the incoherent differential scattering cross section exceeded the sum of the incoherent differential cross sections of the atoms in the sample (Shibayama *et al.*, 2009 ▸; Shibayama *et al.*, 2005 ▸). However, the sum of (dΣ/dΩ)_s-inc_(*Q*) and (dΣ/dΩ)_others_(*Q*) was much smaller than the reported values of 0.89–1.01 cm^−1^ (Shibayama *et al.*, 2009 ▸; Shibayama *et al.*, 2005 ▸) because of the poor sensitivity of the 8 mm-diameter high-angle detector filled with 0.5 MPa ^3^He gas for the inelastically scattered neutrons from the H_2_O sample. Do *et al.* (2014 ▸) and Chen *et al.* (2023 ▸) reported that some of the scattered neutrons from H_2_O gain energies consistent with their thermal energy at room temperature. The fraction of λ = 0.6–0.7 nm neutrons accelerated by the interaction with the 1 mm-thick H_2_O sample was about 33% of the scattered neutrons. The ^3^He tube’s detection efficiency was 88% for the elastically scattered neutrons with λ = 0.65 nm, but only 42% for the inelastically scattered neutrons with the room-temperature thermal energy (λ = 0.17 nm). Even with this non-negligible fraction of accelerated neutrons incident on the spin analyser, (dΣ/dΩ)_others_(*Q*) remains close to zero, as expected.

As shown in Figs. 8 ▸–10 ▸ ▸, the combination of the tight-focusing beam with the remanent supermirror-coated spin analyser enabled polarization analysis measurements. However, the performance of the remanent supermirror-coated spin analyser is still inferior to that of ^3^He spin filters reported elsewhere (Babcock *et al.*, 2013 ▸; Chen *et al.*, 2017 ▸; Gentile *et al.*, 2000 ▸; Chen *et al.*, 2023 ▸; Okudaira *et al.*, 2021 ▸; Okudaira *et al.*, 2020 ▸; Takahashi *et al.*, 2025 ▸). To overcome this limitation and fully exploit the intensity-enhanced tight-focusing polarized beam, we plan to install an *in situ*^3^He spin filter developed at the Materials and Life Science Experimental Facility (MLF), Japan Proton Accelerator Research Complex (J-PARC), at SANS-J. This upgrade will enable high-quality polarization analysis measurements with the tight-focusing polarized beam.

Furthermore, we will apply the tight-focusing polarized beam to spin-contrast-variation SANS (SCV-SANS) measurements, which reveal the structure of composite materials through proton-polarization-dependent polarized neutron scattering (Knop *et al.*, 1991 ▸). Although SCV-SANS measurements were previously performed at SANS-J using a polarized non-focusing beam with a dynamic nuclear polarization apparatus (Kumada *et al.*, 2009 ▸; Kumada *et al.*, 2010 ▸; Noda *et al.*, 2016 ▸), they have more recently been conducted at BL15 (TAIKAN) in J-PARC MLF due to its higher flux of polarized beam (Kumada *et al.*, 2023*b* ▸). By resuming SCV-SANS at SANS-J with the tight-focusing polarized beam, we will establish a complementary experimental environment to J-PARC, thereby highlighting the unique role of SANS-J in advancing polarized neutron scattering studies.

## 4. Conclusion

The use of extended magnetic lenses increased the intensity of the non-polarized beam by a factor of 3.1–6.2 and of the polarized beam by 11.8–21.7, while maintaining or even improving the *Q* resolution for scattering measurements at *Q* > 0.1 nm^−1^. Furthermore, the tight-focusing beam, com­bined with a remanent supermirror-coated spin analyser, enabled separation of coherent and isotope-incoherent scattering from spin-incoherent scattering within 1–4 h, more than an order of magnitude faster than the several days needed with the non-focusing beam. We have already employed these focusing beams for both unpolarized and polarized neutron scattering measurements at *Q* ≥ 0.1 nm^−1^. The loose-focusing beam offers high intensity with excellent *Q* resolution, while the tight-focusing beam provides high intensity together with high neutron polarization, making it particularly effective for scattering measurements of small samples and polarization analysis measurements.

## Figures and Tables

**Table 1:** Calculated *f*, *s*_1_, *s*_2_, 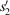, *X*_f_, *A*_f_, ɛ and *P*_n_ for the loose- and tight-focusing beams with *A*_u_ = 20 mm For the loose-focusing beam, *s*_1_ = *s*_1e_, *s*_2_ = *s*_2e_ and 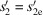 are used. A positive *X*_f_ indicates that the focal point lies downstream of the sample. Beam broadening due to Δλ and gravity (Hammouda & Mildner, 2007 ▸) was not included in *A*_f_, as these contributions are much smaller than (*s*_2_/*s*_1_)*A*_u_ in the present configurations.

								ϕ_s_ = 8 mm		ϕ_s_ = 14 mm	
		*f* (mm)	*s*_1_ (mm)	*s*_2_ (mm)	*s*_2_′ (mm)	*X*_f_ (mm)	*A*_f_ (mm)	ɛ	*P*_n_	ɛ	*P*_n_
Loose-focusing	Horizontal	3140	7790	5260	2238	1700	13.5	3.0	0.88	3.0	0.88
	Vertical		6540	6040	2121	2480	18.5				

Tight-focusing	Horizontal	2400	8365	3370	1865	384	8.1	9.3	0.96	5.2	0.92
	Vertical	2390	7147	3591	1791	638	10.1				

**Table 2:** Intensities (in counts per second, cps), ɛ_p_ and *P*_n_*P*_ana_ of the polarized non-focusing, loose-focusing and tight-focusing beams, measured by the ^3^He high-angle detector placed downstream of the spin analyser The value of *P*_n_ in parentheses was the reported value taken from Oku *et al.* (2007 ▸) for the non-focusing beam (italic) and obtained by substituting *P*_ana_ = 0.87 (*ϕ*_s_ = 8 mm) and *P*_ana_ = 0.89 (*ϕ*_s_ = 14 mm) into *P*_n_*P*_ana_ for the focusing beams. All intensities include a uniform attenuation factor of 1/65.

		φ_s_ = 8 mm			φ_s_ = 14 mm		
		Intensity (cps)	ɛ_p_	*P*_n_*P*_ana_ (*P*_n_)	Intensity (cps)	ɛ_p_	*P*_n_*P*_ana_ (*P*_n_)
Polarized non-focusing	SF off	5.1	–	–	11.2	–	–
	SF on	69. 7	–	–	172.0	–	–
	Total	74.8	1	0.86 (*0.99*)	183.1	1	0.88 (*0.99*)

Loose-focusing	SF off	898.2	–	–	1887.7	–	–
	SF on	162.2	–	–	265.6	–	–
	Total	1060.4	14.2	0.69 (0.81)	2153.3	11.8	0.75 (0.88)

Tight-focusing	SF off	1474.8	–	–	2505.0	–	–
	SF on	146.2	–	–	234.4	–	–
	Total	1621.0	21.7	0.82 (0.95)	2739.4	15.0	0.83 (0.96)

## Data Availability

The data supporting the results can be accessed upon request.
